# Supplementary material for: Neoadjuvant Carboplatin/Paclitaxel versus 5-Fluorouracil/Cisplatin in Combination with Radiotherapy for Locally Advanced Esophageal Squamous Cell Carcinoma: A Multicenter Comparative Study
Source: Cancers (Basel). 2022 May 25;14(11):2610. doi: 10.3390/cancers14112610 (PMC9179264; doi:10.3390/cancers14112610)
Supplement: Supplementary file 1 [file cancers-14-02610-s001.zip › cancers-1701088-supplementary.pdf]

Table S1. Preoperative outcomes of patients in the original cohort

Variable	CROSS (*n* = 124)	PF5040 (*n* = 105)	PF4500 (*n* = 105)	*P* value
Chemotherapy completion (>80%)	111 (89.5)	80 (76)[a]	94 (90)[b]	*.009*
Radiotherapy dose, cGy	4400 ± 161	4964 ± 250[a]	4438 ± 138[b]	*<.001*
Weight loss, %	-1.5 ± 6.0	-2.9 ± 9.0	-3.7 ± 9.2	*.11*
Weight loss >10%	11 (9)	16 (15)	19 (19)	*.081*
Surgical resection rate	92 (74)	77 (73)	70 (67)	*.41*
Reasons for not undergoing surgery				*.52*
Disease progression	13 (11)	14 (13)	14 (13)	*.73*
Patient refusal	11 (9)	11 (11)	14 (13)	*.56*
Poor physical conditions	7 (6)	2 (2)	3 (3)	*.33*
Death during nCRT	1 (1)	1 (1)	4 (4)	*.33*

Abbreviations: nCRT= neoadjuvant chemoradiotherapy

[a]Statistically significant difference *versus* the CROSS group after application of the Bonferroni's correction for multiple comparisons.

[b]Statistically significant difference *versus* the PF5040 group after application of the Bonferroni's correction for multiple comparisons.

Data are presented as means ± standard deviations or counts and percentages, as appropriate.

Table S2. Perioperative outcomes of patients in the original cohort

Variable	CROSS ($n = 92$)	PF5040 ($n = 77$)	PF4500 ($n = 70$)	*P value*
Time from termination of nCRT to surgery, days	64 ± 27	60 ± 22	65 ± 22	*.36*
Thoracic approach				*.82*
Thoracotomy	4 (4)	2 (3)	2 (3)	
Thoracoscopy	88 (96)	75 (97)	68 (97)	
Abdominal approach				*<.001*
Laparotomy	11 (12)	26 (34)[a]	25 (36)[a]	
Laparoscopy	81 (88)	51 (66)[a]	45 (64)[a]	
Type of resection				*.35*
Ivor Lewis	8 (9)	5 (6)	2 (3)	
McKeown	84 (91)	72 (94)	68 (97)	
Postoperative complications				
Anastomotic leak	10 (11)	18 (23)	19 (27)[a]	*.019*
Chylothorax	2 (2)	4 (5)	3 (4)	*.58*
Pulmonary	13 (14)	8 (10)	15 (21)	*.17*
Complication severity (Clavien-Dindo classification)				*.098*
None	40 (44)	38 (49)	22 (31)	
Minor (1-3a)	40 (44)	25 (33)	38 (54)	
Major or death (3b-5)	12 (13)	14 (18)	10 (14)	
Postoperative stay, days	20.7 ± 16.3	23.2 ± 18.6	24.1 ± 17.8	*.45*
30-day mortality rate	3 (3)	2 (4)	2 (3)	*>.99*
T stage				*.011*
T0	31 (34)	39 (51)	26 (37)	
T1	13 (14)	10 (13)	7 (10)	
T2	11 (12)	12 (16)	15 (21)	

T3	36 (39)	14 (18)[a]	16 (23)	
T4	1 (1)	2 (3)	6 (9)	
N stage				*.25*
N0	72 (78)	60 (78)	47 (67)	
N1	15 (16)	14 (18)	16 (23)	
N2	2 (2)	3 (4)	6 (9)	
N3	3 (3)	0(0)	1 (1)	
M stage				*.11*
M0	92 (100)	74 (96)	67 (96)	
M1	0 (0)	3 (4)	3 (4)	
Number of dissected nodes	27.7 ± 13.3	18.2 ± 9.9[a]	22.5 ± 11.2[a]	*<.001*
Pathologically positive nodes	0.66 ± 2.15	0.38 ± 0.93	0.74 ± 1.53	*.36*
ypCR	29 (32)	36 (47)	21 (30)	*.058*
ypT0N+	3 (3)	3 (4)	5 (7)	*.46*
Surgical radicality				*.42*
R0	84 (91)	72 (93)	61 (87)	
R+	8 (9)	5 (7)	9 (13)	
Tumor regression grade				*.016*
TRG1	31 (34)	39 (51)	26 (37)	
TRG2	20 (22)	13 (17)	21 (30)	
TRG3	21 (23)	21 (27)	13 (19)	
TRG4	20 (21)	4 (5)[a]	10 (14)	

Abbreviation: nCRT, neoadjuvant chemoradiotherapy; pCR, pathological complete response; TRG, tumor regression grade

[a]Statistically significant difference *versus* the CROSS group after application of the Bonferroni's correction for multiple comparisons.

Data are presented as means ± standard deviations or counts and percentages, as appropriate.

Table S3. Differences between the current study and the work by Wong et al. [13]

Variable	Current study			Wong et al., 2020	
Study design	Multicenter			Single-center	
Sample size (original cohort)	334			319	
Adjustment for confounding	IPTW of PS			PSM	
Study period (years)	2012–2018	2010–2018	2010–2018	2012–2019	2002–2011
Chemotherapy regimen	CROSS	PF4500	PF5040	CROSS	PF
Radiotherapy dose, Gy	41.4–45	45	50.4	41.4	40
Age, years	57	56	57	65**	64**
cN+ rate	96.6%	93.4%	94%	78%	78%
Tumor length, cm	5.7	5.6	5.5	6**	6**
Progression rate after nCRT	8.8%	11.4%	15.6%	17%	7%
Mortality rate during nCRT	1.5%	2.6%	0.6%	NA	NA
Surgery rate	76.6%	69.2%	72.8%	69%	76%
R0 resection rate	91.1%	87.5%	90.8%	85.5%	81.6%
Number of dissected nodes	27.3	23.0	18.4	36**	33**
pCR rate	29.5%	31.6%	45.3%	24.6%	35.5%
Median survival, months	28.5	20.9	19.5	16.7	32.7

Abbreviations: IPTW inverse probability of treatment weighting; PS, propensity score; PSM, propensity score matching; nCRT, neoadjuvant chemoradiotherapy; pCR, pathological complete response.
Data are presented as means or **medians.
